# 3D Stem Cell Culture

**DOI:** 10.3390/cells9102178

**Published:** 2020-09-27

**Authors:** Joni H. Ylostalo

**Affiliations:** Department of Biology, University of Mary Hardin-Baylor, 900 College Street, Box 8432., Belton, TX 76513, USA; jylostalo@umhb.edu; Tel.: +1-254-295-5534

**Keywords:** stem cell, 3D, culture condition, expansion, niche, regenerative medicine, scaffold, organoid

## Abstract

Much interest has been directed towards stem cells, both in basic and translational research, to understand basic stem cell biology and to develop new therapies for many disorders. In general, stem cells can be cultured with relative ease, however, most common culture methods for stem cells employ 2D techniques using plastic. These cultures do not well represent the stem cell niches in the body, which are delicate microenvironments composed of not only stem cells, but also supporting stromal cells, extracellular matrix, and growth factors. Therefore, researchers and clinicians have been seeking optimal stem cell preparations for basic research and clinical applications, and these might be attainable through 3D culture of stem cells. The 3D cultures recapitulate the in vivo cell-to-cell and cell-to-matrix interactions more effectively, and the cells in 3D cultures exhibit many unique and desirable characteristics. The culture of stem cells in 3D may employ various matrices or scaffolds, in addition to the cells, to support the complex structures. The goal of this Special Issue is to bring together recent research on 3D cultures of various stem cells to increase the basic understanding of stem cells and culture techniques, and also highlight stem cell preparations for possible novel therapeutic applications.

Stem cells are cells that demonstrate the abilities to self-renew and differentiate. Many types of stem cells can be isolated from embryonic or adult tissues, varying in their potency from pluripotent to unipotent depending on the stem cell type. Many cells can be found throughout the human body, often localized in niches that provide a nurturing microenvironment for the stem cells while directing their proliferation and differentiation. Stem cells are typically cultured in 2D tissue culture plastic for ease and maximal expansion, as large numbers are often needed for translational research and therapies. The 2D cultures do not, however, represent the natural environment of stem cells in the body well. Many beneficial properties of stem cells might be lowered or even lost in 2D cultures. Therefore, the use of 3D culture techniques has become more common in basic and translational research. The 3D cultures often provide more complete cell-to-cell and cell-to-matrix interactions, mimicking the natural environment in which the stem cells reside better than the traditional 2D cultures. Furthermore, many desired cellular characteristics are maintained or even promoted in 3D cultures, further supporting their use in basic and translational research. In this Special Issue, recent advances in the 3D cultures of stem cells are highlighted, bringing together a collection of articles presenting various stem cell types and their characteristics in 3D environments.

This Special Issue includes articles highlighting the effective use of induced pluripotent stem cells (iPSCs) to develop cardiac microtissue [1], neurospheres [2], and cortical progenitors [3]. In addition, this Special Issue contains work on scaffolds with mesenchymal stem cells (MSCs) [4] and embryonic stem cells (ESCs) [5]. Furthermore, research on generating intestinal organoids from stem cells [6,7] and nephrogenesis studies utilizing ESCs [8] are included in this Special Issue, along with a comprehensive review on the 3D culture of hematopoietic stem cells (HSCs) [9].

A very relevant problem in cardiology is cardiac fibrosis. In the work by Blyszczuk et al., a model of fibrotic cardiac microtissue was generated using iPSC-derived cardiomyocytes and cardiac fibroblasts by treatment with transforming growth factor β1 (TGF- β1) or by use of cardiac fibroblasts from heart failure patients [1]. The authors demonstrated that activated cardiac fibroblasts could, via direct stimulation of β-adrenoreceptor signaling, promote cardiac contraction rate. Furthermore, the generated model could be used as a high-throughput model for drug testing or general studies of cardiac fibrosis [1].

A 3D in vitro model utilizing iPSCs was generated by Kobolak et al. to study neurotoxicity [2]. In this work, iPSC-derived, 3D, free-floating neurospheres, exhibiting the various cells of the nervous system, were developed and tested in neurotoxicity studies. Furthermore, these neurospheres could open some further opportunities for the detection of developmental neurotoxicity, and hence support the existing animal models [2].

The work by Cutarelli et al. employed iPSC-derived cortical progenitors and scaffolds to mimic the radially oriented cortical radial glia fibers that play an important role in the development of the cerebral cortex [3]. The authors used silicon vertical micropillar arrays to promote the expansion and preservation of stemness of the cortical progenitors. Furthermore, the described model of iPSC-derived cortical progenitors and silicon micropillars could be used in cortical tissue engineering [3].

Another in vitro 3D model was generated by Ciardulli et al. for the study of MSCs and their tenogenic differentiation [4]. This work employed a hyoluronate/poly-lactic-co-glycolic acid (PLGA)/fibrin 3D scaffold with MSCs that were studied under static and cyclic-strain conditions. The research demonstrated that MSCs grown in this scaffold increased their tenogenic marker and pro-repair cytokine expression, supporting the notion of this model as a potential predictive system to be used in future studies employing the sustained release of biochemicals [4].

To circumvent some of the challenges with the 2D culture expansion of ESCs, McKee et al. developed a 3D scaffold to mimic in vivo stem cell niches [5]. The authors used polyethylene glycol (PEG) polymers with thiol and acrylate end-groups to guide the self-assembly of the scaffolds. ESCs grown in these scaffolds were able to maintain their viability, self-renewal, and differentiation potential. ESCs in the scaffolds also exhibited a high expression of pluripotency markers and some mechanosensitive genes, supporting the notion of these scaffolds as potentially helpful for many ESC studies [5].

To aid in the study of intestinal disorders, Kramer et al. developed intestinal organoids from stem cells obtained from various parts of the intestine [6]. These intestinal organoids showed long-term expansion of the cells while inhibiting cell differentiation when cultured in expansion media, while differentiation into goblet and enteroendocrine cells was promoted with culture in differentiation media. These organoids could possibly be employed in various in vitro models of functional intestinal disorders [6].

Another study utilizing intestinal stem cell organoids explored the cellular origin of carcinogenesis [7]. In this study, Yen et al. developed a tumorigenesis model based on carcinogenesis and genetically engineered mice. The authors were able to demonstrate the interplay between extrinsic carcinogen and intrinsic genetic modification and their contribution towards transformation while elucidating the involvement of molecular factors, such as protein phosphatase 2A (PP2A), in this process [7].

A study by Tan et al. utilized ESCs to generate ureteric bud progenitor cells [8]. The authors then used these progenitor cells to induce nephrogenesis in co-culture with primary metanephric mesenchyme. These kidney organoids exhibited nephron structures with collecting ducts connected to nephron tubules. This study demonstrated a relatively simple and reproducible way of generating ureteric bud progenitors [8].

This Special Issue includes articles highlighting recent discoveries in 3D stem cell cultures using MSCs [4], ESCs [5,8], iPSCs [1,2,3], and intestinal stem cells [6,7]. This Special Issue also includes a review article by Ribeiro-Filho et al. that discusses the 2D and 3D culture of hematopoietic stem cells (HSCs) as a relevant model to study both normal and abnormal hematopoiesis [9]. Furthermore, this Special Issue includes articles that utilize various scaffolds together with stem cells from various sources to study 3D cultures [3,4,5].
